# CSPα, a Molecular Co-chaperone Essential for Short and Long-Term Synaptic Maintenance

**DOI:** 10.3389/fnins.2017.00039

**Published:** 2017-02-10

**Authors:** Elena Lopez-Ortega, Rocío Ruiz, Lucia Tabares

**Affiliations:** ^1^Department of Medical Physiology and Biophysics, School of Medicine, University of SevilleSeville, Spain; ^2^Department of Biochemistry and Molecular Biology, School of Pharmacy, University of SevilleSeville, Spain

**Keywords:** cysteine string protein, co-chaperone, motor neurons, synaptic transmission, neuromuscular junction, CSPα

## Abstract

Cysteine string protein α (CSPα) is a vesicle protein located in the presynaptic terminal of most synapses. CSPα is an essential molecular co-chaperone that facilitates the correct folding of proteins and the assembly of the exocytic machinery. The absence of this protein leads to altered neurotransmitter release and neurodegeneration in multiple model systems, from flies to mice. In humans, CSPα mutations are associated with the development of neuronal ceroid lipofuscinosis (NCL), a neurodegenerative disease characterized by intracellular accumulation of lysosomal material. Here, we review the physiological role of CSPα and the pathology resulting from the homozygous deletion of the gene or its mutations. In addition, we investigate whether long-term moderate reduction of the protein produces motor dysfunction. We found that 1-year-old CSPα heterozygous mice display a reduced ability to sustain motor unit recruitment during repetitive stimulation, which indicates that physiological levels of CSPα are required for normal neuromuscular responses in mice and, likely, in humans.

## Protein description

**Figure 1:**
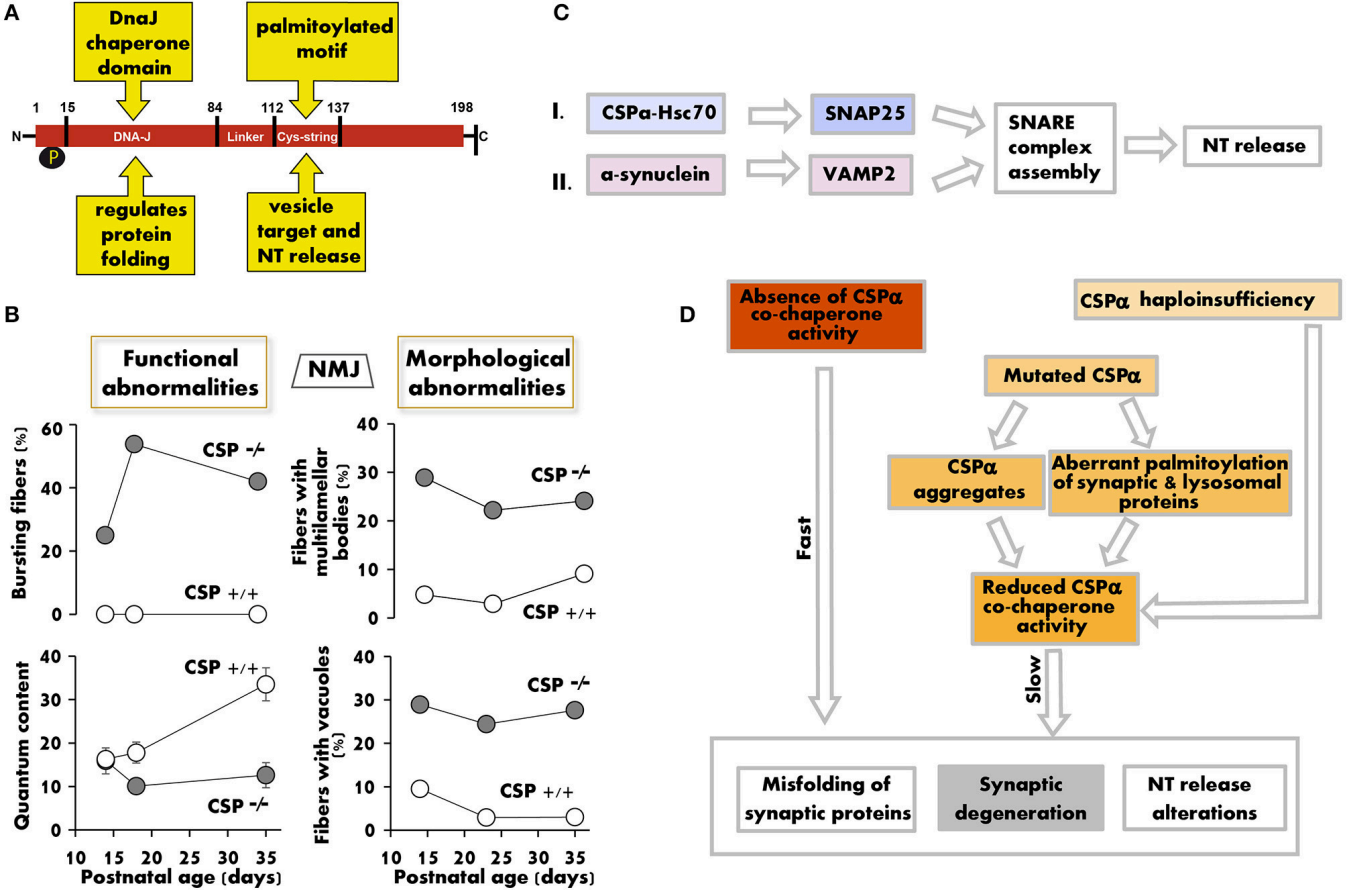
**The lack/decrease of CSPα induces structural and functional changes that compromise synaptic maintenance. (A)** Functional domains of the CSPα protein. **(B)** Synaptic release defects (left) and fraction of motor nerve terminals with multilamellar bodies and vacuoles in electron microscopy profiles (right) vs. postnatal age in CSPα KO mice. Graphs summarize numerical values in Ruiz et al. (2008, 2014) (left) and in Fernández-Chacon et al. (2004) (right). **(C)** Two parallel chaperone pathways promote efficient SNARE complex formation and normal neurotransmission by regulating SNAP25, through CSPα (pathway I), and synaptobrevin (VAMP2), through α-synuclein (pathway II). The deficit in CSPα produces synaptic dysfunction (Ruiz et al., 2008, 2014; Rozas et al., 2012), whose severity, age of onset, and time course depend on the amount of functional CSPα available. α-synuclein overexpression, however, can avoid the synaptic pathology produced by a defect in pathway I by increasing the formation of SNARE complexes (Sharma et al., 2011, 2012a). **(D)** Hypothetical model of how genetic ablation, gene mutations, or increased CSPα degradation induce a positive loop of neurotransmitter (NT) release deficit, accumulation of misfolded synaptic proteins, and neurodegeneration. In humans, it remains unknown to what extent CSPα haploinsufficiency, sequestration of CSPα in aggregates, and aberrant palmitoylation of CSPα and other proteins contribute to ANCL.

Cysteine string protein α (CSPα) (*Dnajc5*) is a highly conserved protein (Figure 1A) typically associated with the membrane of synaptic vesicles and secretory granules (Zinsmaier et al., 1990). It contains a DNA-J domain characteristic of Hsp40 co-chaperones. This domain interacts with the 70 kDa heat shock cognate protein (Hsc70) (Braun et al., 1996) and regulates the refolding of client proteins (Hennessy et al., 2005). A linker region connects the DNA-J domain with the cysteine string domain. The cysteine string domain is approximately 25-amino-acids long and contains 13–15 cysteines, most of them palmitoylated. Palmitoylation is essential to target CSPα to synaptic vesicles and to promote neurotransmitter release (Arnold et al., 2004). CSPα also contains a C-terminal domain, the function of which is not well-understood.

## CSPα deficiency and synaptic dysfunction

CSPα is not essential for synaptogenesis, but it is required for normal neurotransmission and neuronal maintenance in flies (Zinsmaier et al., 1994), worms (Kashyap et al., 2014), mice (Fernández-Chacon et al., 2004), and humans (Nosková et al., 2011). Deletion of the CSPα gene in *Drosophila* produces an embryonic semilethal phenotype, and flies that survive to adulthood present neurotransmitter release alterations and temperature-sensitive paralysis. Synaptic defects in CSPα-null (CSPα KO) mice start early after birth, and death occurs before 3 months of age. Both motor and sensory neurons are affected by the lack of CSPα (Fernández-Chacon et al., 2004; Schmitz et al., 2006). In motor nerve terminals, the first sign of functional alteration appears at 2 weeks of age and consists of repeated bursts of high-frequency spontaneous release (Ruiz et al., 2008) (Figure 1B). Only 4 days later, the terminal displays multiple functional alterations such as reduced quantum content, low release probability, increased short-term facilitation during repetitive stimulation, and reduced calcium sensitivity of the secretory machinery (Ruiz et al., 2008, 2014). In addition, the size of the readily releasable pool of synaptic vesicles is decreased (Rozas et al., 2012; Ruiz et al., 2014).

## CSPα as a molecular co-chaperone

CSPα interacts with several proteins that participate in exo-/endocytosis, including syntaxin, synaptotagmin, N- and P/Q-type calcium channels, and dynamin 1 (for a review see Burgoyne and Morgan, 2015). The functional significance of many of these interactions is not well established. However, one of the best-known functions of CSPα is its role as a co-chaperone (Chamberlain and Burgoyne, 2000; Zinsmaier, 2010; Donnelier and Braun, 2014). CSPα interacts with Hsc70 and together refold client proteins such as SNAP25 (Sharma et al., 2011, 2012a; Zhang et al., 2012). SNAP25 is required for the assembly of the SNARE (soluble N-ethylmaleimide-sensitive factor attachment receptor) complex, formed by synaptobrevin, syntaxin, and SNAP25, which in turn is essential for exocytosis and neurotransmitter release (Figure 1C). In CSPα KO mice, both SNAP25 and SNARE complex levels are reduced by half (Chandra et al., 2005).

## CSPα and neurodegeneration

In CSPα KO motor nerve terminals, hallmarks of degeneration (i.e., vacuoles and multilamellar bodies) appear very early (Fernández-Chacon et al., 2004), even before the evoked release defects become apparent (Figure 1B, left graphs). The degeneration is more prominent in cells with high electrical activity, such as motor neurons, photoreceptors, and GABAergic neurons (Fernández-Chacon et al., 2004; Schmitz et al., 2006; García-Junco-Clemente et al., 2010), suggesting that high synaptic activity potentiates degeneration, which in turn may increase the release deficit (Figure 1D).

At the molecular level, it has been proposed that SNAP25 reduction plays a major role in neurodegeneration. This hypothesis is reinforced by the fact that SNAP25 overexpression in CSPα KO mice prevents neurodegeneration (Sharma et al., 2012a). Nevertheless, SNAP25 heterozygous mice, with 50% of the protein, are phenotypically normal and do not develop neurodegeneration (Washbourne et al., 2002), indicating that solely reducing functional SNAP25 is not sufficient to produce the pathology. On the other hand, misfolded SNAP25 could have a dominant negative effect over the normally folded protein copies, or ubiquitinated SNAP25 molecules could accumulate in the proteasome, interfering with its normal function. Surprisingly, however, the fact that pharmacological inhibition of the proteasome increases SNAP25 and SNARE complex levels, and, hence, improves synaptic function in CSPα-depleted cells (Sharma et al., 2012b), has challenged this last hypothesis.

Remarkably, neurodegeneration in CSPα KO mice is prevented when SNARE complexes are increased by the overexpression of α-synuclein (Sharma et al., 2011, 2012a), in spite of the fact that SNAP25 levels are not restored and, presumably, the amount of misfolded SNAP25 is not reduced. Overexpression of the mutated form of α-synuclein A53T in CSPα KO mice restores life span and motor function as well as wild-type (WT) α-synuclein (Chandra et al., 2005). On the other hand, overexpression of A30P α-synuclein does not rescue survival, but can transitorily ameliorate the release deficit and the calcium sensitivity defect in motor nerve terminals of CSPα KO mice (Ruiz et al., 2014). The partial effect of A30P α-synuclein is likely due to its limited ability to increase the formation of SNARE complexes. These findings suggest that two parallel pathways regulate SNARE complex formation (Figure 1C), and raise the question of how the neurodegeneration program is activated in the absence of CSPα. The molecular basis of the neurodegeneration is unknown, but a possibility is that neurodegeneration is linked to the reduced ability of the synapse to form SNARE complexes and, therefore, to the mismatch between functional demands and efficient release.

## CSPα deficiency in humans

Neuronal Ceroid Lipofuscinosis (NCLs) constitute a heterogeneous group of inherited neurodegenerative disorders characterized by lysosomal accumulation of autofluorescent ceroid-lipofuscin aggregates in neurons and other cell types. The clinical symptoms of NCLs include seizures, movement disorders, cognitive deterioration, and progressive dementia, followed by a premature death. The majority of NCL cases affect children, and only 10% of total cases are in adults.

In recent years, two mutations in the gene that encodes CSPα, *DNAJC5*, have been linked to the development of adult-onset NCL (ANCL) (MIM #162350). These mutations consist of a point mutation (p.L115R) and an in-frame codon deletion (p.L116Δ), both affecting dileucine residues located in the cysteine string domain of CSPα (Nosková et al., 2011). This domain is highly palmitoylated and mediates the membrane binding and intracellular targeting of CSPα. Therefore, mutations in this region may explain the diffuse intracellular localization of CSPα observed in the neurons of ANCL patients. Moreover, the mutated forms of CSPα present an increased tendency to self-associate, forming detergent-resistant aggregates. These aggregates interfere with WT CSPα proteins, reducing the co-chaperone function of CSPα in neurons (Nosková et al., 2011; Greaves et al., 2012; Zhang and Chandra, 2014) (Figure 1D), which is likely one of the main reasons why the NCL-linked *DNAJC5* gene mutations display an autosomal dominant (AD) inheritance pattern. Additionally, a dominant effect of mutated CSPα on the palmitoylation pattern of lysosomal and synaptic proteins has been suggested as a mechanism for the development of *DNAJC5*-linked ANCL (Henderson et al., 2016).

## Long-term moderate CSPα deficiency alters motor responses

**Figure 2:**
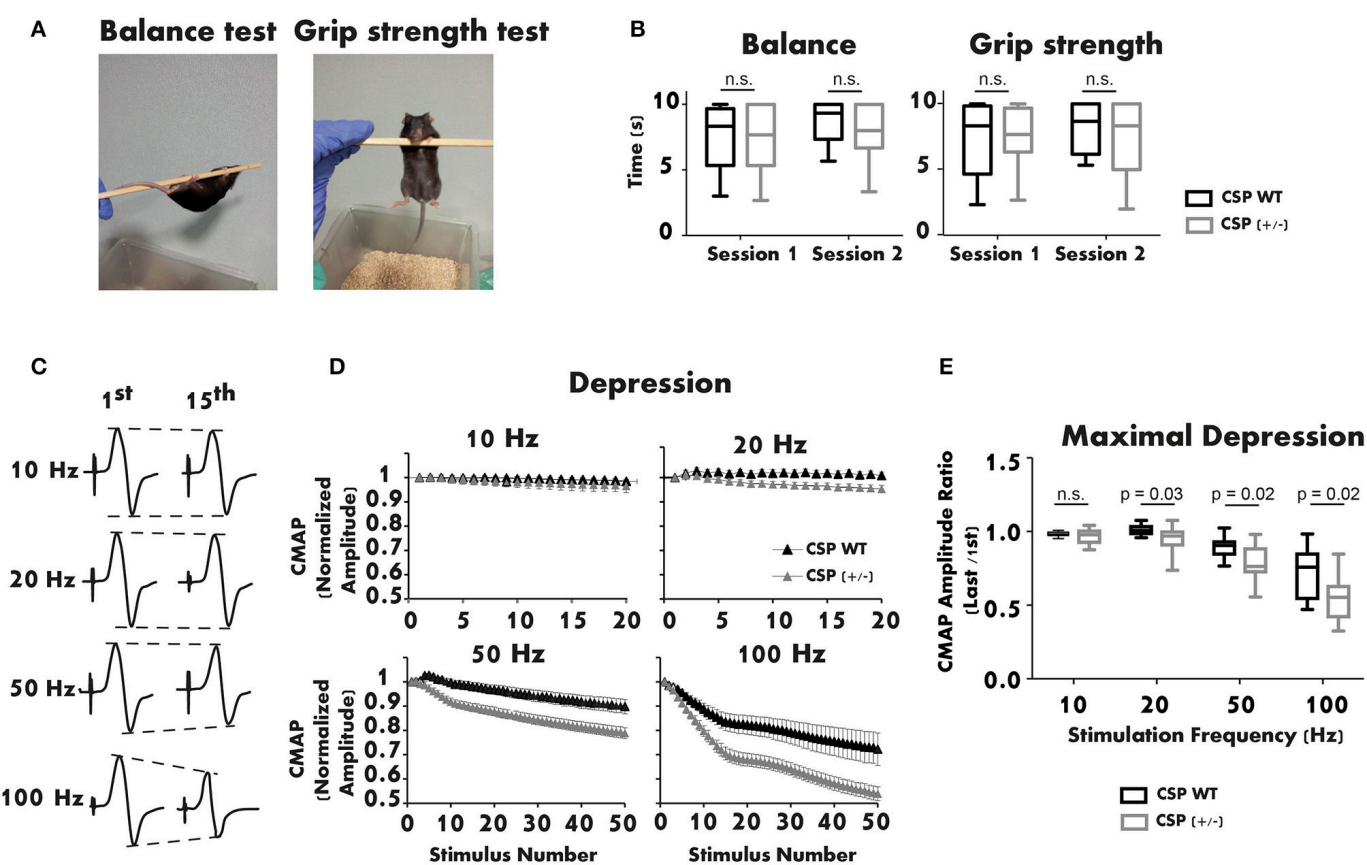
**The neuromuscular function of 1-year-old CSPα heterozygous mice is altered. (A,B)** Balance and grip strength tests show no significant differences between CSPα heterozygous (*n* = 27) and WT mice (*n* = 9). The plot illustrates the average values obtained in three replicates/session for each genotype (Mann-Whitney U test). **(C)** Representative recordings of CMAPs in a CSPα^+/+^ mouse after supramaximal stimulation of the sciatic nerve (1st and 15th responses at different stimulation frequencies). **(D)** Depression of the neuromuscular responses (CMAP normalized amplitude) is significantly larger in CSPα^+/−^ (*n* = 25) than in WT mice (*n* = 7) during stimulation trains at 20, 50, and 100 Hz. **(E)** Maximal depression of the neuromuscular response (normalized) at different stimulation frequencies in CSPα^+/−^ (*n* = 24–25) and WT mice (*n* = 7) (Mann-Whitney U test). Either sex experimental mice (C57BL/6 background) were used. The mouse line was kindly donated by Dr. Südhof. All experiments were performed according to the guidelines of the European Council Directive for the Care of Laboratory Animals. The protocol was approved by the Ethics Committee for Animal Experimentation of the Junta de Andalucía (ref. 23-11-2015-364).

Interestingly, only homozygous CSPα KO mice present synaptic defects, while heterozygous mutant mice appear phenotypically normal up to 3 months of age (Fernández-Chacon et al., 2004). Given the late onset of AD-ANCL (around 30 years of age), we studied the motor function of 1-year-old CSPα heterozygous mice. Motor performance was first assessed with Balance and Grip Strength tests (Figure 2A). The balance was measured by placing the mouse on a horizontal pole suspended in the air. The pole was rotated manually at a constant speed of one rotation cycle per second (Figure 2A left). The grip strength test consisted of suspending the mouse from the pole by its forelimbs (Figure 2A right). In both trials, the amount of time the mouse remained suspended from the pole (maximum 10 s) was measured. Two sessions separated by 1 week were performed for each test (Figure 2B). Data obtained from the neurological tests showed no significant differences between WT and CSPα heterozygous mice, in either balance or grip strength.

Next, the neuromuscular response of CSPα heterozygous mice was studied using electromyography (EMG). Evoked Compound Motor Action Potentials (CMAPs) were recorded from the right lateral gastrocnemius of anesthetized mice. Stimulation needle electrodes were placed at the sciatic notch and the head of the fibula (Ruiz et al., 2005). The active recording electrode was placed in the medial region of the recorded muscle. The reference electrode was inserted at the base of the fifth foot phalanx. The ground electrode was located at the base of the tail. Brief supramaximal stimulation pulses were applied at 10 Hz (2 s), 20 Hz (1 s), 50 Hz (1 s), and 100 Hz (0.5 s). Representative recordings of CMAPs registered during the trains are shown in Figure 2C. The study revealed enhanced depression with stimulations between 20 and 100 Hz in CSPα heterozygous mice compared to WT littermates (Figure 2D). The mean maximal depression was 5% (20 Hz), 12% (50 Hz), and 25% (100 Hz) lower in CSPα heterozygous than in WT mice, while no significant difference was observed at 10 Hz (Figure 2E). Remarkably, the depression in the EMG recordings was similar to that seen in 3-week-old CSPα KO mice (Fernández-Chacon et al., 2004), a phenotype not observed in CSPα^+/−^ mice up to 3 month of age. These results indicate that, over time, a moderate reduction of CSPα expression alters the ability of the neuromuscular system to respond normally to stimulation.

## Future directions

The multiple functions of CSPα range from acting as a chaperone, participating in the assembly and dissociation of multi-protein complexes, and regulating Ca^2+^ sensitivity for neurotransmitter release. The severe functional and structural changes that take place in the absence of CSPα in invertebrate and vertebrate organism models confirm the importance of this protein in synapse maintenance and neurotransmitter release. In humans, CSPα mutations are associated with the development of AD-ANCL, synaptic degeneration, and neuronal loss. Therefore, although both the reduction of CSPα expression and the presence of CSPα mutations are pathogenic to the synapse, the severity and time course of the neurological impairments may vary from severe, including premature death, to mild, depending on the amount of functional CSPα in each case. The moderate decrease in CSPα and SNARE complexes in neurons over time could result in motor function impairment and, in addition, influence the evolution of common age-related neurodegenerative disorders, such as Alzheimer's and Parkinson's diseases. Future challenges are to identify patients with reduced levels of molecular chaperones (such as CSPα), decipher the mechanisms responsible for the molecular deficit, understand how the homeostasis of the synapse is altered, and determine to what extent the reduction of the chaperones influences the severity of associated neurodegenerative diseases.

## Author contributions

Experiments shown in Figure 2 were performed by EL. EL, RR, and LT conceived and wrote the manuscript.

## Funding

This work was supported by grants from the Spanish Ministry of Science and Innovation (BFU2013–43763-P) and the Tatiana Perez de Guzman Foundation to LT. RR was supported by a contract from the *V Plan Propio* of the University of Seville.

### Conflict of interest statement

The authors declare that the research was conducted in the absence of any commercial or financial relationships that could be construed as a potential conflict of interest.
